# *catena*-Poly[[(1,10-phenanthroline-κ^2^ *N*,*N*′)copper(I)]-μ_2_-iodido]

**DOI:** 10.1107/S1600536810043205

**Published:** 2010-10-31

**Authors:** Yisheng Yang, Wenxiang Chai, Li Song, Kangying Shu

**Affiliations:** a College of Materials Science and Engineering, China Jiliang University, Hangzhou 310018, People’s Republic of China; b Department of Chemistry, Key Laboratory of Advanced Textile Materials and Manufacturing Technology of Education Ministry, Zhejiang University of Science and Technology, Hangzhou 310018, People’s Republic of China

## Abstract

The solvothermal reaction of copper(I) iodide and 1,10-phenanthroline (phen) in ethanol yielded the title polymeric compound, [CuI(C_12_H_8_N_2_)]_*n*_. The asymmmetric unit comprises one Cu^+^ cation, one I^−^ anion and one phen ligand. Each Cu^+^ cation is in a distorted tetrahedral coordination by two iodide anions and two N atoms from a bidentate chelating phen ligand. The Cu^+^ cations are bridged through the iodide anions, leading to a zigzag chain structure extending parallel to [100]. There are π–π inter­actions among adjacent phen ligands of one chain [centroid–centroid distance = 3.693 (3) Å].

## Related literature

For other copper(I)–iodide complexes with 1,10-phenanthroline as a co-ligand, see: Healy *et al.* (1985 ▶); Yu *et al.* (2001 ▶, 2002 ▶, 2004 ▶); Zhou *et al.* (2005 ▶); Zhang *et al.* (2008 ▶).
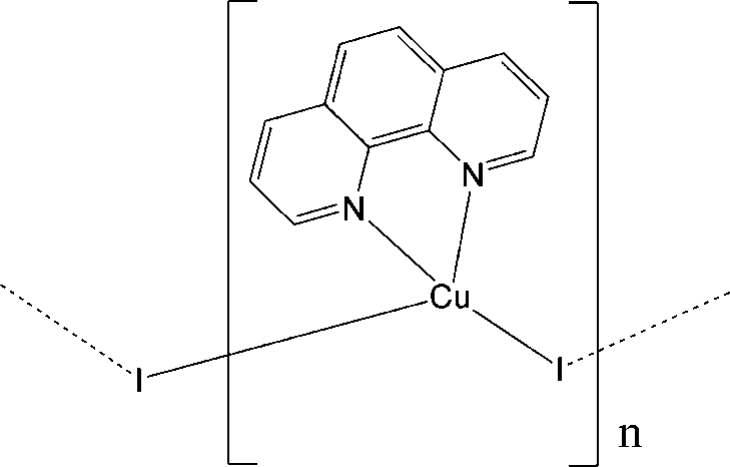


## Experimental

### 

#### Crystal data

[CuI(C_12_H_8_N_2_)]
*M* *_r_* = 370.64
Orthorhombic, 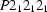

*a* = 4.1664 (5) Å
*b* = 10.4621 (11) Å
*c* = 25.518 (4) Å
*V* = 1112.3 (2) Å^3^
*Z* = 4
Mo *K*α radiation
μ = 4.71 mm^−1^
*T* = 293 K
0.35 × 0.10 × 0.05 mm

#### Data collection

Rigaku R-AXIS RAPID diffractometer
Absorption correction: multi-scan (*ABSCOR*; Higashi, 1995 ▶) *T*_min_ = 0.290, *T*_max_ = 0.799
8582 measured reflections
2567 independent reflections
2380 reflections with *I* > 2σ(*I*)
*R*_int_ = 0.027

#### Refinement

*R*[*F* ^2^ > 2σ(*F* ^2^)] = 0.028
*wR*(*F* ^2^) = 0.056
*S* = 1.12
2567 reflections
145 parameters
H-atom parameters constrained
Δρ_max_ = 0.94 e Å^−3^
Δρ_min_ = −0.54 e Å^−3^
Absolute structure: Flack (1983 ▶), 1018 Friedel pairs
Flack parameter: 0.05 (3)

### 

Data collection: *PROCESS-AUTO* (Rigaku, 1998 ▶); cell refinement: *PROCESS-AUTO*; data reduction: *CrystalStructure* (Rigaku/MSC, 2004 ▶); program(s) used to solve structure: *SHELXS97* (Sheldrick, 2008 ▶); program(s) used to refine structure: *SHELXL97* (Sheldrick, 2008 ▶); molecular graphics: *SHELXTL* (Sheldrick, 2008 ▶); software used to prepare material for publication: *SHELXTL*.

## Supplementary Material

Crystal structure: contains datablocks I, global. DOI: 10.1107/S1600536810043205/kp2280sup1.cif

Structure factors: contains datablocks I. DOI: 10.1107/S1600536810043205/kp2280Isup2.hkl

Additional supplementary materials:  crystallographic information; 3D view; checkCIF report

## Figures and Tables

**Table 1:** Selected bond lengths (Å)

I1—Cu1	2.5895 (6)
I1—Cu1^i^	2.6030 (6)
Cu1—N2	2.100 (3)
Cu1—N1	2.110 (3)

Symmetry code: (i) 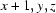.

**Fig. 1.**
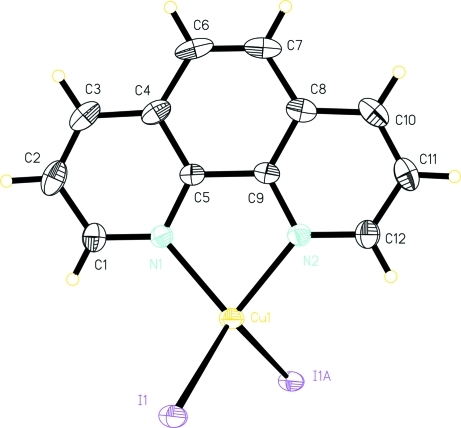
Structure and labelling of the title compound, with displacement ellipsoids drawn at the 30% probability level and H atoms shown as small spheres of arbitrary radii. Symmetry code A: x - 1, y, z.

**Fig. 2.**
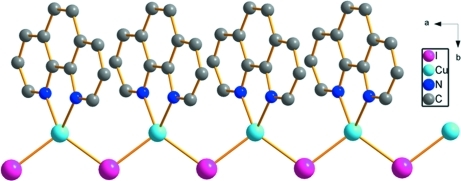
The chain structure constructed from Cu(phen)I unit.

**Fig. 3.**
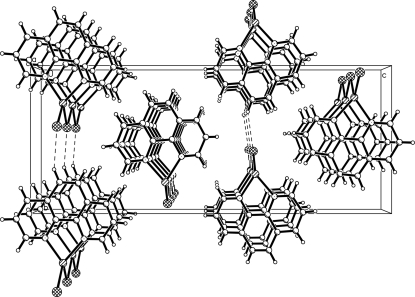
The packing diagram viewed along the a-direction.

## supplementary crystallographic information

### Comment

Recently, there have been a number of reports of copper(I)-iodide complexes with 1,10-phenanthroline (phen) as a coligand. Among them, almost all complexes are disctete molecules (Healy *et al.*, 1985; Yu *et al.*, 2001; Yu *et al.*, 2002; Yu *et al.*, 2004; Zhou *et al.*, 2005; Zhang *et al.*, 2008) except two complexes characterized as polymeric structure (Zhang *et al.*, 2008). We have synthesised the polymeric title complex [Cu(phen)I]_n_ (I) (Fig.1). It is worthy of note that compound I crystallizes in a noncentrosymmetric space group of *P*2_1_2_1_2_1_, while the other copper(I)-iodide complexes with phen all crystallise in centrosymmetric space groups. The asymmmetric unit contains one Cu^+^ cation, one I^-^ anion and one phen ligand. Each Cu^+^ cation is tetrahedrally coordinated by two iodide anion and two nitrogen atoms from a bidentate chelating phen ligand. The Cu^+^ cations are bridged through the iodide anions, leading to a zigzag chain structure. The Cu—I bond lengths are 2.5895 (6) and 2.6030 (6) Å, which are similar to that found in other copper(I)-iodide complexes. There are π-π interactions between adjacent phen ligands of one chain. The phen skeletons are arranged in a perfect parallel fashion with centroid-centroid distance of 3.693 (3) Å (from two adjacent C4/C5/C6/C7/C8/C9 ring and C1A/C2A/C3A/C4A/C5A/N1A ring, symmetry code A: *x* - 1, *y*, *z*).

### Experimental

All chemicals were obtained from commercial sources and were used as received. The title compound was handily synthesized by a solvothermal reaction from CuI and phen. A mixture of Cu^I^ (76 mg, 0.4 mmol) and phen.H_2_O (80 mg, 0.4 mmol) in 12 mL alcohol was put into a Parr Teflon-lined autoclave (23 mL) and heated at 393 K for 3 days. After cooling down to room temperature, yellow crystals of compound I were obtained.

### Refinement

The structure was solved using direct methods and refined by full-matrix least-squares techniques. All non-hydrogen atoms were assigned anisotropic displacement parameters in the refinement. All hydrogen atoms were added at calculated positions and refined using a riding model. (Sheldrick, 2008).

### Crystal data

**Table:**

[CuI(C_12_H_8_N_2_)]	*F*(000) = 704
*M**_r_* = 370.64	*D*_x_ = 2.213 Mg m^−^^3^
Orthorhombic, *P*2_1_2_1_2_1_	Mo *K*α radiation, λ = 0.71075 Å
Hall symbol: P 2ac 2ab	Cell parameters from 2969 reflections
*a* = 4.1664 (5) Å	θ = 2.1–27.5°
*b* = 10.4621 (11) Å	µ = 4.71 mm^−^^1^
*c* = 25.518 (4) Å	*T* = 293 K
*V* = 1112.3 (2) Å^3^	Prism, yellow
*Z* = 4	0.35 × 0.10 × 0.05 mm

### Data collection

**Table:**

Rigaku R-AXIS RAPID diffractometer	2567 independent reflections
Radiation source: fine-focus sealed tube	2380 reflections with *I* > 2σ(*I*)
graphite	*R*_int_ = 0.027
Detector resolution: 14.6306 pixels mm^-1^	θ_max_ = 27.5°, θ_min_ = 2.5°
CCD_Profile_fitting scans	*h* = −5→5
Absorption correction: multi-scan (*ABSCOR*; Higashi, 1995)	*k* = −13→13
*T*_min_ = 0.290, *T*_max_ = 0.799	*l* = −33→31
8582 measured reflections	

### Refinement

**Table:**

Refinement on *F*^2^	Secondary atom site location: difference Fourier map
Least-squares matrix: full	Hydrogen site location: inferred from neighbouring sites
*R*[*F*^2^ > 2σ(*F*^2^)] = 0.028	H-atom parameters constrained
*wR*(*F*^2^) = 0.056	*w* = 1/[σ^2^(*F*_o_^2^) + (0.0192*P*)^2^ + 0.4704*P*] where *P* = (*F*_o_^2^ + 2*F*_c_^2^)/3
*S* = 1.12	(Δ/σ)_max_ = 0.001
2567 reflections	Δρ_max_ = 0.94 e Å^−^^3^
145 parameters	Δρ_min_ = −0.53 e Å^−^^3^
0 restraints	Absolute structure: Flack (1983), 1020 Friedel pairs?
Primary atom site location: structure-invariant direct methods	Flack parameter: 0.05 (3)

### Special details

**Table:**

Geometry. All e.s.d.'s (except the e.s.d. in the dihedral angle between two l.s. planes) are estimated using the full covariance matrix. The cell e.s.d.'s are taken into account individually in the estimation of e.s.d.'s in distances, angles and torsion angles; correlations between e.s.d.'s in cell parameters are only used when they are defined by crystal symmetry. An approximate (isotropic) treatment of cell e.s.d.'s is used for estimating e.s.d.'s involving l.s. planes.
Refinement. Refinement of *F*^2^ against ALL reflections. The weighted *R*-factor *wR* and goodness of fit *S* are based on *F*^2^, conventional *R*-factors *R* are based on *F*, with *F* set to zero for negative *F*^2^. The threshold expression of *F*^2^ > σ(*F*^2^) is used only for calculating *R*-factors(gt) *etc*. and is not relevant to the choice of reflections for refinement. *R*-factors based on *F*^2^ are statistically about twice as large as those based on *F*, and *R*- factors based on ALL data will be even larger.

### Fractional atomic coordinates and isotropic or equivalent isotropic displacement parameters (Å^2^)

**Table:**

	*x*	*y*	*z*	*U*_iso_*/*U*_eq_	
I1	0.66039 (6)	0.42090 (2)	0.117804 (11)	0.03947 (8)	
Cu1	0.16241 (14)	0.27290 (4)	0.12007 (2)	0.03758 (12)	
N1	0.3102 (9)	0.1092 (3)	0.07823 (11)	0.0338 (7)	
N2	−0.0053 (9)	0.1283 (3)	0.17018 (13)	0.0341 (7)	
C5	0.2334 (9)	−0.0008 (4)	0.10317 (14)	0.0315 (9)	
C12	−0.1751 (12)	0.1378 (4)	0.21386 (15)	0.0436 (10)	
H12	−0.2174	0.2191	0.2269	0.052*	
C9	0.0515 (10)	0.0084 (4)	0.15161 (15)	0.0315 (9)	
C4	0.3219 (12)	−0.1225 (4)	0.08441 (16)	0.0420 (10)	
C1	0.4834 (11)	0.1005 (5)	0.03491 (16)	0.0448 (11)	
H1	0.5378	0.1752	0.0173	0.054*	
C8	−0.0525 (10)	−0.1027 (4)	0.17715 (17)	0.0419 (11)	
C10	−0.2305 (11)	−0.0865 (5)	0.22338 (17)	0.0536 (12)	
H10	−0.3048	−0.1575	0.2417	0.064*	
C6	0.2110 (14)	−0.2340 (4)	0.1123 (2)	0.0557 (14)	
H6	0.2677	−0.3146	0.1000	0.067*	
C11	−0.2940 (12)	0.0332 (5)	0.24137 (17)	0.0532 (12)	
H11	−0.4150	0.0450	0.2716	0.064*	
C3	0.5059 (13)	−0.1268 (5)	0.03889 (19)	0.0519 (13)	
H3	0.5720	−0.2050	0.0254	0.062*	
C7	0.0286 (14)	−0.2250 (4)	0.1555 (2)	0.0546 (14)	
H7	−0.0462	−0.2990	0.1715	0.065*	
C2	0.5884 (12)	−0.0168 (5)	0.01436 (18)	0.0546 (14)	
H2	0.7135	−0.0189	−0.0158	0.066*	

### Atomic displacement parameters (Å^2^)

**Table:**

	*U*^11^	*U*^22^	*U*^33^	*U*^12^	*U*^13^	*U*^23^
I1	0.02550 (13)	0.02861 (12)	0.06429 (16)	0.00102 (12)	0.00327 (14)	0.00180 (12)
Cu1	0.0322 (3)	0.0280 (2)	0.0525 (3)	0.0003 (2)	0.0052 (3)	0.0017 (2)
N1	0.0326 (18)	0.0320 (17)	0.0367 (15)	0.0001 (16)	0.0003 (16)	−0.0017 (12)
N2	0.0335 (18)	0.0320 (16)	0.0368 (18)	−0.0010 (16)	−0.0010 (16)	−0.0011 (14)
C5	0.026 (2)	0.0296 (18)	0.0385 (19)	−0.0005 (14)	−0.0077 (17)	0.0004 (14)
C12	0.037 (2)	0.052 (2)	0.042 (2)	0.001 (3)	0.001 (2)	−0.0009 (18)
C9	0.024 (2)	0.0300 (19)	0.040 (2)	−0.0022 (15)	−0.0092 (18)	0.0041 (16)
C4	0.036 (2)	0.038 (2)	0.052 (2)	0.010 (2)	−0.015 (2)	−0.0114 (18)
C1	0.039 (2)	0.055 (3)	0.041 (2)	0.003 (2)	0.0016 (19)	−0.001 (2)
C8	0.036 (2)	0.038 (2)	0.052 (2)	−0.0063 (19)	−0.0157 (19)	0.0050 (19)
C10	0.046 (3)	0.059 (3)	0.056 (3)	−0.012 (3)	−0.002 (2)	0.022 (2)
C6	0.060 (3)	0.027 (2)	0.080 (3)	0.009 (2)	−0.029 (3)	−0.012 (2)
C11	0.042 (3)	0.073 (3)	0.045 (2)	−0.005 (3)	0.006 (2)	0.012 (2)
C3	0.046 (3)	0.052 (3)	0.059 (3)	0.014 (3)	−0.013 (3)	−0.023 (2)
C7	0.058 (3)	0.029 (2)	0.076 (3)	−0.005 (2)	−0.024 (3)	0.009 (2)
C2	0.043 (3)	0.077 (4)	0.044 (2)	0.013 (3)	−0.005 (2)	−0.020 (2)

### Geometric parameters (Å, °)

**Table:**

I1—Cu1	2.5895 (6)	C4—C6	1.443 (6)
I1—Cu1^i^	2.6030 (6)	C1—C2	1.404 (6)
Cu1—N2	2.100 (3)	C1—H1	0.9300
Cu1—N1	2.110 (3)	C8—C10	1.404 (6)
Cu1—I1^ii^	2.6030 (6)	C8—C7	1.434 (6)
N1—C1	1.323 (5)	C10—C11	1.360 (7)
N1—C5	1.354 (5)	C10—H10	0.9300
N2—C12	1.324 (5)	C6—C7	1.341 (7)
N2—C9	1.361 (5)	C6—H6	0.9300
C5—C4	1.409 (5)	C11—H11	0.9300
C5—C9	1.453 (5)	C3—C2	1.355 (7)
C12—C11	1.391 (6)	C3—H3	0.9300
C12—H12	0.9300	C7—H7	0.9300
C9—C8	1.402 (5)	C2—H2	0.9300
C4—C3	1.392 (7)		
			
Cu1—I1—Cu1^i^	106.715 (18)	N1—C1—C2	122.9 (4)
N2—Cu1—N1	79.66 (13)	N1—C1—H1	118.6
N2—Cu1—I1	135.30 (10)	C2—C1—H1	118.6
N1—Cu1—I1	103.90 (10)	C9—C8—C10	117.0 (4)
N2—Cu1—I1^ii^	100.05 (10)	C9—C8—C7	119.2 (4)
N1—Cu1—I1^ii^	134.92 (10)	C10—C8—C7	123.8 (4)
I1—Cu1—I1^ii^	106.715 (18)	C11—C10—C8	119.8 (4)
C1—N1—C5	117.6 (3)	C11—C10—H10	120.1
C1—N1—Cu1	129.6 (3)	C8—C10—H10	120.1
C5—N1—Cu1	112.6 (2)	C7—C6—C4	122.0 (4)
C12—N2—C9	117.1 (3)	C7—C6—H6	119.0
C12—N2—Cu1	129.5 (3)	C4—C6—H6	119.0
C9—N2—Cu1	113.2 (3)	C10—C11—C12	119.0 (4)
N1—C5—C4	123.2 (3)	C10—C11—H11	120.5
N1—C5—C9	117.8 (3)	C12—C11—H11	120.5
C4—C5—C9	119.1 (4)	C2—C3—C4	119.9 (4)
N2—C12—C11	123.8 (4)	C2—C3—H3	120.1
N2—C12—H12	118.1	C4—C3—H3	120.1
C11—C12—H12	118.1	C6—C7—C8	120.9 (4)
N2—C9—C8	123.3 (4)	C6—C7—H7	119.6
N2—C9—C5	116.6 (3)	C8—C7—H7	119.6
C8—C9—C5	120.1 (4)	C3—C2—C1	119.4 (4)
C3—C4—C5	117.1 (4)	C3—C2—H2	120.3
C3—C4—C6	124.2 (4)	C1—C2—H2	120.3
C5—C4—C6	118.6 (4)		

Symmetry codes: (i) *x*+1, *y*, *z*; (ii) *x*−1, *y*, *z*.
